# Methods for Assessing Spillover in Network-Based Studies of HIV/AIDS Prevention among People Who Use Drugs

**DOI:** 10.3390/pathogens12020326

**Published:** 2023-02-15

**Authors:** Ashley L. Buchanan, Natallia Katenka, Youjin Lee, Jing Wu, Katerina Pantavou, Samuel R. Friedman, M. Elizabeth Halloran, Brandon D. L. Marshall, Laura Forastiere, Georgios K. Nikolopoulos

**Affiliations:** 1 Department of Pharmacy Practice, University of Rhode Island, Kingston, RI 02881, USA; 2 Department of Computer Science and Statistics, University of Rhode Island, Kingston, RI 02881, USA; 3 Department of Biostatistics, Brown University, Providence, RI 02912, USA; 4 Medical School, University of Cyprus, 1678 Nicosia, Cyprus; 5 Department of Population Health, New York University, New York, NY 10016, USA; 6 Vaccine and Infectious Diseases Division, Fred Hutchinson Cancer Center, Seattle, WA 98109, USA; 7 Department of Biostatistics, University of Washington, Seattle, WA 98195, USA; 8 Department of Epidemiology, Brown University School of Public Health, Providence, RI 02912, USA; 9 Department of Biostatistics, Yale School of Public Health, New Haven, CT 06520, USA

**Keywords:** spillover effects, networks, people who use/inject drugs, human immunodeficiency virus

## Abstract

Human Immunodeficiency Virus (HIV) interventions among people who use drugs (PWUD) often have spillover, also known as interference or dissemination, which occurs when one participant’s exposure affects another participant’s outcome. PWUD are often members of networks defined by social, sexual, and drug-use partnerships and their receipt of interventions can affect other members in their network. For example, HIV interventions with possible spillover include educational training about HIV risk reduction, pre-exposure prophylaxis, or treatment as prevention. In turn, intervention effects frequently depend on the network structure, and intervention coverage levels and spillover can occur even if not measured in a study, possibly resulting in an underestimation of intervention effects. Recent methodological approaches were developed to assess spillover in the context of network-based studies. This tutorial provides an overview of different study designs for network-based studies and related methodological approaches for assessing spillover in each design. We also provide an overview of other important methodological issues in network studies, including causal influence in networks and missing data. Finally, we highlight applications of different designs and methods from studies of PWUD and conclude with an illustrative example from the Transmission Reduction Intervention Project (TRIP) in Athens, Greece.

## 1. Introduction

People who use drugs (PWUD) remain vulnerable to infectious diseases, such as HIV, hepatitis C (HCV), and other sexually transmitted infections [1] through both injection and sexual transmission routes [2]. Injection drug use, however, is the pivotal dimension of the HIV and HCV epidemics among PWUD. A systematic review in 2017 estimated that the global burden of harm associated with drug use impacted around 15.6 million people who inject drugs (PWID), with many of them being infected by HIV (17.8%) or having antibodies to HCV (52.3%) [3].

Consideration beyond individual risk behaviors for the prevention of HIV transmission is required because people are often members of networks [4,5,6,7,8]. Networks are defined as groups of individuals connected through sexual, drug, social, or other relationships. Since the early 1990s, there has been increasingly rigorous research evaluating the role of individuals’ relationships, including those associated with HIV risk, and of the structure of social networks in health and disease in general and in HIV infection in particular [9,10,11,12,13,14]. For instance, the pioneering work in Colorado Springs in the early 1990s showed that the structure of the network of high-risk people could explain, to some extent, why the prevalence of HIV was paradoxically low in their population [15]: HIV-infected people were in the periphery of the network, usually forming small groups. Social network-based studies in New York City in the 1990s and in Athens, Greece, in the 2010s have shown that HIV prevalence is significantly higher in the densest parts of the PWID networks. Moreover, the configuration of these networks—for example, the k-cores, which are subsets of a network component whose members are connected with at least k other subset members—was associated with drug injection-related norms; that is, what people believe others do (descriptive norms) or their perception of how others react (approve or disapprove) to their behavior (injunctive norms) [16,17]. For example, a safe behavioral pattern, such as the discouragement of using injection equipment that is not sterile, was less normative among contacts of people in the two-core compartment of the network than among network contacts of people outside the two-core [17].

HIV research among PWUD might utilize network information in several important ways. First, we can study the associations of the network features with participant characteristics to understand how HIV risk behaviors, such as sharing injection drug equipment, are related to network density or centrality [18]. Common network metrics to understand the structure include density, degree, and centrality [19,20,21]. There is also extensive literature on statistical modeling approaches in networks to quantify associational parameters that model the network structure itself or features of the study participants [22,23,24,25]. We can compare HIV risk networks with other types of networks including social, sexual, and drug use networks to better understand the role of these types of networks in relation to HIV transmission risk [26]. We can also leverage networks to increase enrollment in studies involving marginalized populations such as PWUD. For example, we can improve the delivery of interventions by locating individuals recently infected with HIV in the network [27,28,29,30].

Research has also been trying to shed light on how the social context and its role in perpetuating or reducing engagement in HIV risk behavior and/or care could help plan and deliver better HIV interventions among PWID [6,31,32,33,34,35,36]. For example, a network-based intervention (Transmission Reduction Intervention Project—TRIP) used as “seeds” (study participants to begin network tracing) people who had recently acquired HIV to recruit more recently infected individuals, and refer them to treatment, as contacts of those recently infected are more likely to be infected themselves. This network-based intervention was successfully implemented in Athens, Greece; Chicago, United States; and Odessa, Ukraine. The network-based intervention contributed to HIV prevention and improved medical care for the participants of that project [37,38]. While behavioral and biomedical interventions reduce individual HIV acquisition risk, the development of network-based interventions has the potential for substantial benefit beyond the exposed individual. For example, treatment as prevention (TasP) could be delivered as a network intervention by providing TasP along HIV risk recruitment chains to recently infected individuals. Although these approaches are essential for understanding HIV transmission risk to develop HIV interventions, the potential benefit of interventions beyond the treated individuals is largely missed. In fact, it is likely that this intervention not only improves the disease status of treated HIV-positive individuals, but also prevents onward transmission to HIV-negative partners [37].

When one participant’s exposure affects another participant’s health outcome, the effect is broadly known as spillover (alternatively, interference or dissemination) [39]. Spillover has been well studied to improve vaccine delivery and effectiveness in a population [40,41,42]; however, there have been limited evaluations of spillover for HIV interventions [43]. Spillover can generally exist in a network when an intervention is delivered to individuals connected through disease transmission risk or some other mechanism [44,45,46]. Beyond individual interventions in networks, network-based interventions are a way to leverage networks both for targeted treatment delivery and increasing spillover effects [47,48]. Evaluation of spillover among first-degree contacts in networks may be more relevant for HIV-related interventions than other types of spillover set (i.e., groups of individuals for which spillover is possible) definitions, such as those based on the distance in the network from the person receiving the intervention, because HIV transmission usually occurs through direct contact, sexual or parenteral [39]. Spillover with infectious disease outcomes can be further disentangled into contagion (i.e., when one participant’s outcome affects another participant’s outcome), peer effects (i.e., when one participant’s outcome affects their contact’s future outcomes), and infectiousness (i.e., when an intervention renders a participant less likely to transmit the disease to another participant) [49,50,51]. In this tutorial, we focus on the spillover of individual interventions delivered in networks and network-based interventions more broadly without consideration of the underlying disease mechanisms.

Despite the increasing volume of studies and the expansion of social network-based research in the fields of HIV transmission and prevention [9], the limitations of existing methodological tools for network data analyses in general and for studying spillover effects in particular prevent a complete evaluation of the impact of these interventions and limit the information obtained from network-based studies [39]. Importantly, spillover effects can occur even in HIV research that does not measure networks, and PWUD are nonetheless part of networks, and thus spillover should not be ignored. In fact, ignoring the underlying network and spillover effects of interventions reduces the quality of such research and the interventions informed by that research. Moreover, the current period, with the globe having been seriously affected by another infectious disease (SARS-CoV-2) and the associated policies to address it, is characterized by the rapid collection of a large amount of data, including those related to network contacts of infected individuals. It is thus crucial to understand the challenges and opportunities related to research on networks, specifically networks among PWUD. In addition, new methodological tools for analyzing network data have been recently developed, allowing for assessments beyond the simple descriptive methods borrowed from graph theory and the more sophisticated inferential approaches to address the problem of dependence between units in networks [52,53,54,55]. These new methodological approaches now allow researchers and public health professionals to reveal and understand the health impacts of HIV-related and other interventions via evaluation of spillover effects. Here, we explain recent developments in causal inference methods to estimate spillover effects in a non-technical language, refer the reader to methodological references for complete technical details, present examples from studies that assessed spillover of HIV-related network-based interventions among PWUD, and then conclude with a discussion of crucial future research in this area. Moving from randomized experiments to observational studies, we describe each network-based study design, the identifying assumptions, and the methods proposed in the literature.

## 2. Causal Spillover in Network-Based Studies

In HIV network-based studies, individuals (known as nodes) are typically connected through sexual relationships, substance-use behaviors, or social relationships (known as connections, links, or edges). These individuals are recruited and enrolled in the study, and information on the connections between study participants is ascertained. In marginalized and/or structurally vulnerable populations, individuals may be recruited through respondent-driven sampling (RDS) or snowball sampling, where participants then recruit additional participants, or through vertex sampling, such as single or multiple waves of contact tracing. In some studies, known as sociometric or full network studies, the connections are then ascertained (to the extent possible) between all recruited participants. Some studies have focused on a closed population, such as a school, village, or community. Participants may be recruited into these full network studies through RDS. The recruitment edges (or ties) are ascertained by asking the recruit about connections to the recruiter, and information about connections to other individuals is ascertained similarly to the initial recruitment prompts. In addition, there is a growing literature on randomized designs in networks; however, these randomized designs may not be ethical for HIV interventions, such as pre-exposure prophylaxis (PrEP) or TasP. Therefore, recent methods were developed for nonrandomized interventions to assess spillover in network-based studies [51,56,57,58]. We briefly outline network-based study designs and related statistical methodology among PWUD and provide examples of each design from the literature. We also refer the readers to Friedman et al. (2018) [59] for an overview of other risk and social network methods among PWUD.

When evaluating the effects of non-randomized interventions, causal inference to assess spillover in the context of networks can be challenging. The use of a potential outcomes framework provides an approach to deal with well-understood biases, such as confounding and selection bias, and address complex dependencies in outcomes due to the network structure [56,57,58], as well as biases specific to network designs, including homophily [60] and network dependence [61,62]. Using a potential outcomes framework, we can index each participant’s potential outcome by their own exposure and the exposure of others in the network. This means that each participant’s exposure and the exposures of other participants in the network could possibly affect that participant’s outcome, and each person has many (potential) outcomes. For now, we consider static interventions, which set the intervention status to a particular realization and do not depend on an individual’s characteristics [51]. With appropriate identification assumptions, contrasts of average observed (potential) outcomes can yield estimates of causal effects.

**Figure 1:**
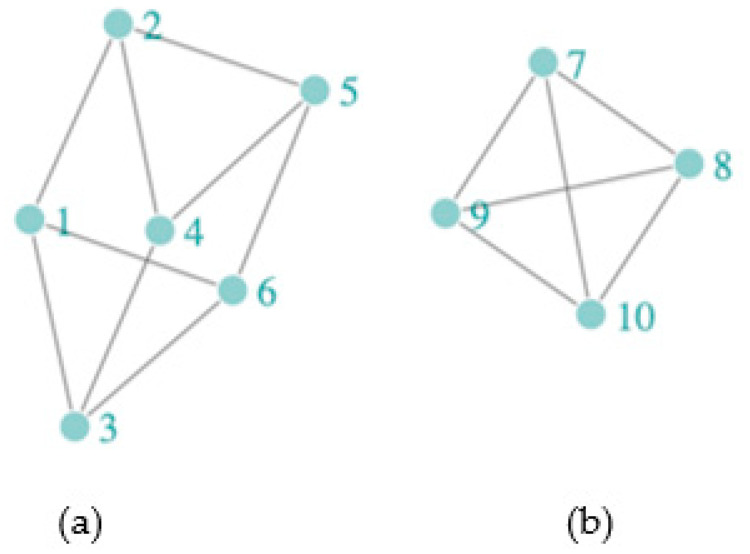
A sample network with two components, (**a**) Component 1 = {1, 2, 3, 4, 5, 6} and (**b**) Component 2 = {7, 8, 9, 10}, with participants (nodes) denoted by light-green circles and connections (edges) denoted by gray lines. The nearest neighbors of participant (node) 2 are {1, 4, 5}, of participant (node) 3 are {1, 4, 6}, and of participant (node) 6 are {1, 3, 5}.

Assessing spillover requires definitions for the spillover set and often also an exposure mapping. The spillover set for each participant is defined as the group of individuals whose treatment (or exposure) may affect the participant’s (potential) outcome. In the context of network studies, spillover set definitions can include connected components or subnetworks (i.e., groups of individuals connected by at least one path but not connected to others in the network). Alternatively, spillover sets can be defined to include the entire network or nearest-neighbor sets defined, for example, by first-degree contacts in the observed network [46]. Using components as the spillover set relies on a partial interference assumption, which assumes that the spillover occurs only among individuals within the component but not between components, and non-overlapping (i.e., unconnected) components can be defined in the network. Figure 1 displays the structure of a sample network with two components. If we assumed the spillover sets comprised the (non-overlapping) components, then spillover would be possible between all participants in each component, but not between the two components. On the other hand, if we assumed the spillover set included only the nearest neighbors (i.e., first-degree neighbors defined by the observed edges), the spillover set for participant 2 includes participants 1, 4, and 5. This is known as the neighbor interference assumption [63]. Note that for the neighbor interference assumption, the participants’ spillover sets can overlap. In contrast, the case with the spillover set defined as the component defines the spillover sets as non-overlapping sets of participants. A broader approach allows for a generalized spillover set, where a grouping of other participants is the spillover set unique to each participant. We can also consider partial interference and neighbor interference to be special cases of a generalized spillover set [64,65]. By exposure mapping, we mean how the exposures of others in the spillover set are related to the potential outcomes of each participant in the study. One commonly used approach to defining the exposure mapping, known as the stratified interference assumption, assumes that a participant’s outcome depends on exposures of those in the spillover set through a summative function, such as the total number or proportion exposed [66,67]. We require that the spillover set and exposure mapping are correctly specified to quantify the spillover effect of interest.

**Figure 2:**
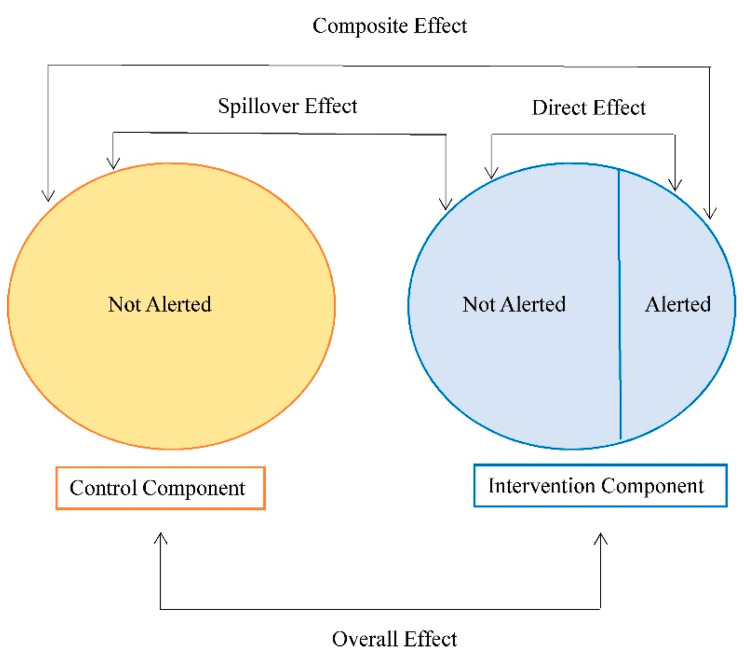
Parameters of interest in the presence of spillover in a network-based study. The exposure in this example is the receipt of a community alert intervention about a possible increased risk of HIV acquisition in the network. Adapted from [72].

Different causal parameters can be defined when spillover is present to assess intervention effects. Commonly considered parameters include the direct (individual), spillover (indirect), composite (total), and overall effects (Figure 2). These effects typically differ from mediation, which uses similar terminology for direct and indirect effects, and assesses the role of a meditating variable on the causal effect [68]. The parameters related to spillover can be defined by comparing expected (average) potential outcomes under counterfactual scenarios defined by the individual assignment to intervention/treatment, and the treatment allocation strategy in the spillover set [45,69]. The treatment allocation strategy defines the probability of assignment to intervention or treatment for the group (e.g., those in the spillover set and the index individual or the spillover set only). If this probability of assignment in the spillover set does not depend on covariates, then it is referred to as intervention coverage in the literature [57,70]. We refer to receipt of the intervention or treatment for individual participants as exposure (versus no exposure). The direct effect contrasts average (potential) outcomes under exposure versus no exposure with a fixed intervention coverage level. The spillover effect contrasts average (potential) outcomes if a participant was not exposed, comparing two intervention coverage levels. The composite effect is a function of both the direct and spillover effects and is a measure of the maximal intervention effect, comparing average (potential) outcomes under exposure with a higher intervention coverage level to no exposure with a lower intervention coverage level. Lastly, the overall effect marginalizes over the participant-level exposure and compares two different treatment allocations for the group. Alternatively, parameters can be defined as expectations across the entire network, for example, contrasting the average potential outcomes under one treatment allocation for the entire network versus no treatment in the network [51,71], or expectations that fix the exposure for participant i and set the exposures for the neighbors to the observed values [56]. Table 1 summarizes the definitions of these effects generally for network-based studies. Other definitions of these effects may be possible.

Several assumptions are needed to assess these causal effects in a network-based study. Exchangeability, positivity, and treatment-variation irrelevance assumptions for causal inference under the potential outcomes framework are required, and we discuss each of these assumptions specific to this setting [73,74]. When the intervention is not randomized, we assume conditional exchangeability for the exposure of both the individual and their spillover set, which allows for the identification of causal contrasts related to both the individual’s exposure and the treatment allocation strategy for their spillover set [56,57,58]. We assume positivity holds; that is, participants and individuals in the spillover sets are exposed (and not exposed) for each level of the covariates. Treatment-variation irrelevance implies we have only one version of being exposed to the intervention and only one version of not being exposed, which allows the potential outcomes to correspond to specific intervention exposures. Assumptions about the spillover set and possibly also the exposure mapping specific to the study intervention [57] are also required. For example, one could assume that an individual’s outcome is only influenced by the number of their first-degree neighbors who are exposed, which focuses locally in the network to define the spillover set and may be appropriate for some HIV interventions. Additionally, one may assume that the (potential) outcomes depend on the number exposed in the spillover set, rather than who specifically was exposed (i.e., stratified interference). Additional assumptions are required specific to each study design, which are described in more detail below.

## 3. Network-Based Study Designs and Methods

The problem of spillover (or interference) has been well-studied in studies with clustering features (i.e., groups of individuals). Numerous causal inference methods are available to relax the no interference (spillover) assumption and allow for interference within but not between clusters; this is called partial interference [67,75,76,77,78]. With this assumption, a grouping of individuals (e.g., households, villages) can be used to define the spillover set; however, connections or partnerships (i.e., edges) between individuals in the group are either not measured or utilized in the causal inference method, and individuals cannot be in more than one group [64]. Recent methodological advances provide new approaches to assess spillover among PWUD in network-based studies. In particular, methods incorporating the connections in the observed network structure were proposed to better understand the spillover of interventions. We discuss the study design, identifying assumptions, and methods for each type of network-based study organized by study type. The study design should be selected based on the research question, ethics, and feasibility, among other considerations. We begin with describing randomized designs in networks because these designs can provide strong protection against confounding bias, then discuss approaches for observational designs when randomization is not ethical or feasible. We describe ego-network randomized designs, which only randomize index participants to an intervention. Then, we describe two additional approaches to study networks that often lack a randomized intervention: respondent-driven samples and sociometric networks.

### 3.1. Randomized Designs in Networks

In a two-stage randomized design [69,79,80], groups of individuals are assigned to an allocation strategy, then according to that allocation strategy, individuals in a group are assigned to intervention (or not) according to their group allocation strategy. This randomized design allows for the evaluation of spillover with fewer assumptions than an observational study. In this case, a partial interference assumption is made; that is, there is possibly spillover between individuals within a group, but not between groups. Although two-stage randomized trials have been conducted among PWUD [81], this study design has limited applications in network settings with groups defined by components insofar as we are aware. Depending on the spillover set definition considered in the network, this design may be feasible for network-based studies among PWUD. For example, in a network with the spillover set defined as the entire component, components could be first randomized to an intervention allocation strategy, then individuals within the component could be randomized to intervention (or no intervention). This may require multiple study sites to ensure enough components for valid inference in the study. However, if the spillover set is defined as the set of nearest neighbors [57,65], this two-stage design may not be feasible because the spillover sets could be overlapping and no longer comprise unique individuals. Therefore, the allocation strategies for each group would likely depend on the strategies of other groups. Ordering exposure or treatment assignments across nearest-neighbor sets may be possible, but the design would be fairly complex and challenging to implement in the real world. In two-stage randomized designs, estimators for the individual- and group-weighted parameters were developed [79]. With two-stage randomization, unbiased estimators of the suite of effects have been developed. Assuming stratified inteference, corresponding variance estimators were shown to be unbiased under an additional assumption and conservative otherwise [69,79]. In a cluster-randomized trial (e.g., groups of participants are randomized to an intervention or not), if group size is informative, individual- and group-weighted parameters may be different, so specifying the parameter of interest is important, as well as ensuring the estimation approach is appropriate [82]. In cluster-randomized trials, the overall effects and estimators could be defined using contrasts of cluster-level outcomes, while the spillover could be defined using individual-level outcomes [83]. For example, the spillover effect could be defined as the effect of the intervention on individuals who do not participate in the trial. The contrast could be defined as the average outcomes among non-participants when under cluster intervention versus no cluster intervention. This effect can be defined and estimated using a principal stratification approach [83]. Furthermore, a direct effect could be estimated by adjusting for measured confounders, and if we assume trial participation does not depend on randomization, a spillover effect can also be assessed [83,84]. In addition, some definitions of the direct effect in clustered studies may not capture the actual individual effect of the intervention under certain randomizations when outcomes are contagious [85]. Another approach develops bounds for a complier average causal effect (i.e., effect among those who complied with the assigned intervention) in a cluster-randomized trial with both spillover and non-compliance [84].

Recent advances in randomized designs allow for the evaluation of spillover in networks [86,87,88,89,90]. Much of this work is motivated by studies of social media with interest in studying the diffusion of information and knowledge. There have been few applications of this design to studies among networks of PWUD insofar as we are aware. In the context of HIV prevention and treatment interventions, randomization of the intervention may not be ethical or feasible. In the randomized design, participants in the network can be randomly assigned to an intervention (or no intervention), and the (potential) outcome(s) for each participant depends on the intervention assignments to others in the network, which results in many potential outcomes. Assumptions and conditions are required to asess spillover effects in this setting by reducing the total number of (potential) outcomes. One approach uses exposure mappings [64], which reduces the number of possible exposures for an individual. For example, one could consider the exposures for each participant’s first- or second-degree neighbors to define the exposure mapping. The participants used to define the exposure mapping would be those individuals in the spillover set. With this reduction in possible exposures, one can assess the (potential) outcome when the participant is exposed, and the other participants in the spillover set are exposed (or not); on the other hand, when the participant is not exposed the other participants in the spillover set are exposed (or not). Various contrasts of estimators of these quantities can be defined to assess spillover and related effects. The approach to treatment assignment is an important consideration in the study design and should align with network exposure mapping. For example, suppose each participant is assigned a Bernoulli probability p of treatment, and the exposure mapping for an individual’s contacts is reduced to at least one contact exposed (versus no contacts exposed). In that case, the probability of each exposure mapping can be calculated. For example, the probability of participant i themselves not being treated along with their first-degree neighbors not being treated is ${\left(1-p\right)}^{d_{i}+1}$, where $d_{i}$ is the degree for participant i. Based on this, the individual and joint exposure probabilities can be calculated for each of the four possible exposures (e.g., the individual and at least one participant in the spillover set is exposed; only the individual is exposed; the individual is not exposed and at least one participant in the spillover set is exposed; or no exposure for the individual or participants in the spillover set) [64]. In this randomized design, one approach to estimate spillover effects is a Horvitz–Thompson type estimator with unequal probability sampling with inverse probability weighted (IPW) estimators, and conservative variance estimators have been proposed [64]. Although randomization provides protection against confounding, randomized designs can be vulnerable to other issues, including generalizability [91], measurement error of spillover sets [92,93], non-compliance [84], and selection bias due to differential loss to follow-up [94,95], that warrant further consideration when evaluating spillover (see Section 3.5.2).

#### Extensions to Observational Studies

When randomization is not feasible or ethical, observational studies can be conducted to assess spillover. Extensions to two-stage IPW estimators have been developed for an observational setting evaluating a nonrandomized intervention with partial interference defined by clusters or groups of individuals [45,77], including stabilized IPW estimators that possibly improve efficiency [65], causal estimands that allow for within-cluster dependence [96,97], and estimators that are doubly robust (i.e., either the outcome model or propensity score needs to be correctly modeled for valid inference) [98]. These weighted estimators are group-level averages of the outcome with weights defined as the inverse probability of exposure for the group, which can be estimated using a mixed effects logit model [45,77]. In a network-based study, these estimators could assume partial interference with groups defined by the network components. Although we likely cannot conduct two-stage randomization in a network, we could define parameters based on a treatment allocation strategy in a network, and employ estimators similar to those used in observational studies.

### 3.2. Ego-Network Randomized Designs

An ego-network randomized study is defined as a study where index participants are recruited from the population (through random or non-random sampling) and randomized to the intervention, while their egocentric network (i.e., first-degree contacts with connections only to the index) is also ascertained [44,46,99]. While the intervention is only randomly assigned to the index participants, baseline and follow-up measures are collected for both the index participants and their network members. Typically, there is only one index participant in each network. The index participants are sometimes referred to as “egos” and the network members are called “alters”. In an egocentric-network randomized study, the spillover (or interference) set could be conceptualized in two different ways: (1) groups defined by ego-networks (ignoring connections or edge information) or (2) ego-network structures (using edge information). Determining which is appropriate for a particular study relies on the assumptions about the exposure spillover mechanism and the role of index status.

Often in the literature, these studies are analyzed as if the design was a cluster-randomized trial [100,101], and overall effects are assessed with the exposure defined as the group-level intervention. Behavioral interventions have been developed to reduce HIV transmission through both injection and sexual routes of transmission [1,102]. Several studies suggest that peer-led interventions aimed at HIV prevention among PWUD in networks can increase the beneficial effects [100,101,103,104,105]. For example, one approach trains PWUD to educate members of their existing egocentric HIV risk networks (defined as groups of individuals possibly with sexual or injection risk behavior partnerships), as well as ways to reduce their injection risk behavior. These interventions may be successful because peers are community members and may educate and influence their network members to modify their risk behaviors [106]. Peer-led interventions have been demonstrated to reduce injection-related HIV risk behaviors compared to HIV testing and counseling alone [100,101,103,104,105]. These studies often focus on overall effects defined by a comparison of outcomes among intervention and control networks [101], evaluation of associations of network-level characteristics with outcomes [107], or unadjusted comparisons of outcomes index and network members [48]. These approaches can assess causal overall effects and associations in the study; however, additional considerations are needed in this setting to assess causal spillover effects. Several methods have been developed that go beyond the overall effect to assess direct and spillover effects [44]. Different considerations may be needed when studying the spillover of social and informational interventions compared to the spillover of biomedical interventions. For example, in the context of informational training to reduce HIV risk behaviors, the training could result in all members of the intervention group changing their knowledge and possibly their behavior, which suggests that considering the group-level intervention status in addition to the individual-level intervention status (i.e., peer educator) may be important.

**Figure 3:**
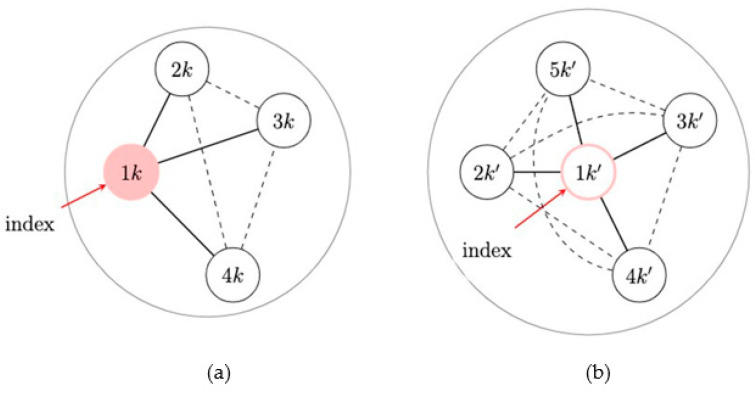
Neighbor interference assumption in an ego-network randomized design. (**a**) An intervention network: the network with an exposed index member. (**b**) A network without intervention: the network with an unexposed index. Black lines indicate that the edges are used in the approach and dashed lines indicate that the edges may be present but are not used in the approach.

The first consideration concerns the definition of spillover sets. A reasonable assumption is that the spillover set for index participants coincides with their egocentric network. This assumption implies that index participants cannot be affected by the intervention delivered to other index participants, which is reasonable given that index participants are likely not connected. In addition, this assumption implies that index participants cannot be affected through unobserved connections between their network members and network members of other intervention index participants. This consideration is often plausible. In fact, although we collect the egocentric networks of index participants in an ego-network randomized study, the relationship between network members of different index participants is unknown. However, we can assume that the degree of separation between egocentric networks is enough to rule out a cascade in behavioral changes through different egocentric networks. For example, due to the study design with an intervention that trains the index to educate their network members only, we could assume that network members are only connected to one index participant. These considerations also imply that spillover sets of network members do not involve participants belonging to different networks. If the assumption of no spillover between ego networks is a concern, studies may be able to collect information to assess contamination from the intervention to control networks [108], such as intervention information recall, or ensure the networks are disparate enough to reduce contamination. However, the definition of spillover sets for network members requires more consideration. In fact, we typically do not ascertain information on the connection between network members of the same index participant, and network members can also be connected to individuals not in the sample. Nevertheless, because neither out-of-sample individuals nor network members can receive the intervention, if we assume that it does not matter who receives the intervention in the ego network, a version of the stratified interference assumption [69], then spillover sets of network members can be said to coincide with their index participant and their egocentric network. In this setting and under these considerations, this version of the neighbor interference assumption actually corresponds to the partial interference assumption, which assumes that the entire ego network is the spillover set for all participants of that ego network [47]; that is, there is spillover possibly between all participants within an ego network but not between ego networks (Figure 3). Under this assumption, we describe two possible frameworks for evaluating spillover in ego-network randomized designs.

The first approach assumes that each participant’s (potential) outcome(s) can be affected by their own exposure and the number of exposed individuals in their spillover set, and this is known as the neighbor interference assumption (with a stratified interference assumption). This neighbor interference assumption coincides with a partial interference assumption in this setting [56]. In an ego-network randomized design, an index participant can either receive the intervention (or not), but their network members in the spillover set are always unexposed to the intervention; on the other hand, network members cannot be exposed to the intervention, but they can have one exposed individual in their spillover set—the index participant. This approach considers a set of parameters for an ego-network randomized design. The direct effect is defined as the effect of receiving the intervention, and this can be estimated by comparing intervention index members to everyone in control networks. The spillover effect is defined as the effect of being unexposed to the intervention while being connected to an exposed individual (versus not), and this can be estimated by comparing network members whose index is exposed to the intervention to everyone in control networks. Due to the randomization of the ego to intervention (or not), the estimation in this setting does not require adjustment for measured baseline variables to control for confounding. Thus, estimation can be achieved by using a difference-in-means estimator, contrasting the average outcomes among network members whose index is exposed versus not for spillover effects, and among exposed-versus-unexposed index participants for direct effects. This approach assumes no differences between the ego and alters at baseline in the absence of intervention, which could be problematic if the ego self-selects to serve in this role. If this self-selection is a concern, then the comparison group for the estimation of the direct effect would only include unexposed index participants, while the comparison group for the estimation of the spillover effect would only include network members of unexposed index participants. In this procedure, direct and spillover effects are only estimated for index participants or network members, respectively, and methods to generalize such effects are required (e.g., inverse probability of index selection).

For the second approach in egocentric-network randomized studies, partial interference is still assumed; however, the role of index participants is investigated more explicitly, while the difference between index participants and network members is adjusted as part of the estimation procedure in terms of their pre-exposure baseline covariates [44]. This approach mimics an ideal two-stage randomized design, where the groups defined by the ego-networks are randomly assigned to an intervention (or not), and one network member is randomly assigned to be an index in each ego-network. To mimic this design, (potential) outcomes are indexed by the index status and the intervention received by the group, including the index participant and their egocentric network. In this approach, spillover is not defined separately for indexes and network members, but assumed to be all captured by an ego-network-level randomization variable (i.e., intervention or control) [44]. In this design, groups are often followed longitudinally over time to allow for a possible shift in network norms [47], which results in the intervention group possibly being different from the control group in terms of their group-level exposure status and (potential) outcomes. For example, training is received by the index, then the index trains their network members. At this point, the index and network members are exposed to the study intervention (i.e., information about reducing risk behavior). They can also share that information with other network members and even reinforce knowledge of their index peer educator or affect their index through behavioral change. This renders membership in the intervention group an important part of the intervention exposure in addition to the individual-level exposure (e.g., index status). In this second setting, a set of parameters are defined in the presence of spillover [44]. The spillover effect is defined as the effect of being an unexposed network member in an intervention network with an exposed index (versus in a control network with an unexposed index), and this can be estimated by comparing network members in intervention networks to network members in control networks. The direct effect is defined as the effect of being an exposed index oneself beyond being in an intervention network. This can be estimated by comparing index members to network members in intervention networks. In this approach, estimation requires adjustment for measured baseline variables that may differ between the index and network members. Possible estimation approaches include generalized estimating equations or generalized linear mixed models that allow the analyst to also account for the correlation of individual outcomes in the ego networks [44]. While in this second approach, this effect of reinforcement through network norms is captured by the both the direct and spillover effects, in the first approach, it is captured by the direct effect, which combines the effect of receiving intervention for index participants and reinforcement from network members.

**Figure 4:**
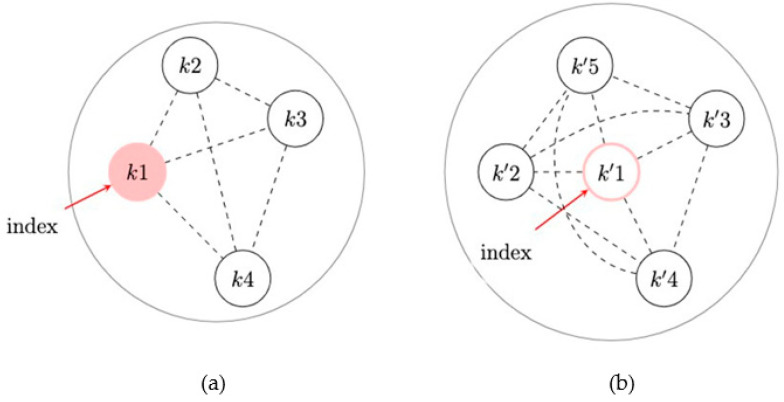
Partial interference assumption with a focus on index status in an ego-network randomized design. (**a**) An intervention network: the network with an exposed index member. (**b**) A network without intervention: the network with an unexposed index member. Dashed lines indicate that the edges may be present but are not used in the approach.

Recent work developed approaches to assess spillover in ego-network randomized designs. Buchanan et al. (2018) proposed a method using generalized estimating equations assuming the spillover set is the index and their egocentric network (Figure 4) [44,99]. The direct effect is the effect for index members beyond being in an intervention network; the spillover effect is the effect for network members who possibly receive the intervention benefit from their exposed index member. Although the overall effect estimator is valid due to the network randomization, Buchanan et al. (2018) demonstrated that the overall effect estimates will generally be less than the composite effect estimates. To illustrate the usefulness of this method in real-world data, Buchanan et al. (2018) analyzed the HIV Prevention Trials Network 037 Study, a phase III, network-level, randomized controlled HIV prevention trial. This study was conducted in the United States and Thailand from 2002 to 2006 and recruited people who inject drugs, and participants were followed for up to 30 months to assess risk behavior. Index participants were assigned to either an intervention group (peer educator) or a control group, and their risk network members were not peer educators themselves, but likely interacted with their index participant who was a peer educator [44]. In the model adjusted for baseline variables, we expect a 35% rate reduction in the report of risk behaviors if a participant is an index member in an intervention network versus if a participant is a network member in a control network (rate ratio = 0.65, 95% confidence interval: 0.47, 0.90).

### 3.3. Respondent-Driven Samples

The respondent-driven sample (RDS) framework was developed by Douglas Heckathorn (1997) [109] and RDS sampling usually follows methods from Salganik and Heckathorn (2004) [110]. This approach assumes these individuals are part of drug-use, injection, and sexual networks and begins with a convenience sample of participants (known as seeds). Then, participants share coupons with their contacts, who can then be recruited into the study until the appropriate sample size is achieved. Compared to snowball sampling (begins with a convenience sample, then all relevant contacts for each person are sampled), RDS could result in less dependency between participants if individuals recruited into the study are further from the initial seeds due to a fixed sample size.

Most statistical inference approaches for RDS estimate population proportions as binary or categorical covariates. In terms of statistical inference in RDS, sampling often depends on convenience samples for the initial seeds, network contacts, and with whom participants decide to share their coupons, rendering model approaches for these features a challenge. For example, RDS sampling has been approximated using random walks [110], first-order Markov sampling [109], and successive sampling [111]. Different network structures and sampling assumptions are required for estimation. In addition, RDS typically only provides partial information about the true underlying network. We refer the reader to this review of RDS sampling and methods and these additional papers for recent methods development in RDS, including a bivariate inference approach in RDS [112,113,114]. Evaluation of spillover in RDS remains challenging due to the sample not representing the full network [115,116], and methods in the evaluation of spillover in networks often require the network to be known. Furthermore, multivariable methods for RDS remain limited [117], and adjustment for confounders is often a prerequisite for causal inference for interventions lacking randomization. In Yauck, et al. (2021) [117], a generalized mixed-effects model with spatial random effects for the seeds or recruiters and homophily terms (to account for similar individuals sharing edges) can also include RDS weights to conduct inference in the population. We recommend additional future work that leverages approaches to understand the full network derived from an RDS sample to assess spillover in the full network [118,119,120], as well as additional advancements in multivariable methods in RDS to allow for adjustment for covariates to address confounding.

RDS is often used to recruit PWUD into a study to estimate population proportions (e.g., the prevalence of diseases) and population size [121,122,123]. For example, HIV, HCV, and STI Risk Associated with Nonmedical Use of Prescription Opioids (NMU-PO) was a mixed methods study [36,124] evaluating drug use and sex-related HIV/HCV/STI (sexually transmitted infections) risk associated with NMU-PO obtained through a RDS among 539 young adults in the New York City area, including people who engage in injection drug use and heroin use. The study team aimed to identify individual, network, and socio-structural factors that increase the risk of injection drug use in this population. Twenty eligible individuals with NMU-PO within the past 30 days were recruited as RDS seeds to initiate the recruitment chain of their contacts who also use opioids. Data collection was completed in 2015. In their primary analysis, the investigators employed the successive sampling estimator [111] to obtain RDS-adjusted population estimates. There was a high prevalence of nonmedical benzodiazepine use among study participants (93%), and polysubstance use, drug binging, heroin injection, and overdose were associated with regular nonmedical benzodiazepine use [124]. In addition, about 20% of study participants were hepatitis C (HCV) antibody-positive [125], and knowing a person who uses opioids and is 29 years or older was associated with testing positive for HCV [126].

### 3.4. Sociometric Networks

Causal inference methodologies to assess spillover among PWUD in network-based studies have recently been developed. These methods consider the observed edges in the network and relax the partial interference assumption by no longer requiring non-overlapping groups of individuals in the network (i.e., partial interference). Approaches using chain graphs were developed to provide parsimonious representations of network data, including a model for outcomes associated with nodes with dependence along the edges due to social interactions, contagion, or interference [127]; direct transmission and latent characteristics [51]; and another approach [71] using an auto-g-computation algorithm, which is a version of Robins’ g-formula adapted to networks [128]. Forastiere et al. (2021) [56] and Lee et al. (2022) [57] allowed for overlapping spillover sets by using spatial proximity or network ties to define the groups. In Liu et al. (2016) [65], an approach using an IPW estimator was developed that also allows for overlap of the spillover sets by assuming a generalized spillover set (i.e., a unique spillover set for each participant); however, the variance estimator assumes partial interference defined by larger groups of study participants. For demonstration, we highlight three estimation approaches below for sociometric networks with non-randomized interventions that also have publicly-available software to conduct the analysis.

In Forastiere et al. (2021) [56], a subclassification estimator with a generalized propensity score was developed to assess the effects of nonrandomized interventions in networks under neighbor interference. This paper relies on the unconfoundedness property of a joint propensity score and the propensity of the observed individuals’ and neighbors’ exposures. Thanks to this property, the covariate imbalance between units exposed to different individuals’ and neighbors’ exposures can be adjusted for in the analysis by conditioning on a joint propensity score. A factorization of a joint propensity score into an individual and a neighbor propensity score is used to separate the joint propensity score-based adjustment into individual and neighbor terms. After identifying subclasses with similar individual propensity scores, estimation proceeds by fitting an outcome regression conditional on the neighbor propensity score within each subclass. To estimate the variance, a bootstrapping procedure with resampling at the individual or group level was proposed, using groups of individuals or communities, such as schools, villages, or network components identified by community detection algorithms [56]. This approach uses a joint propensity score for both an individual and their neighbor. The propensity score is described in more detail below as the second IPW, where the estimation approach uses inverse probability weights instead of subclassification. In addition to the development of a generalized propensity score-based estimator, an important contribution of Forastiere et al. (2021) [56] is the derivation of the bias that a naïve estimator of the intervention effect would experience when spillover is not considered.

**Figure 5:**
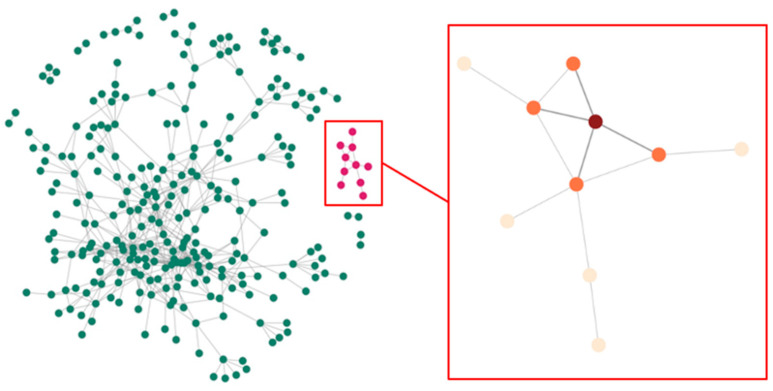
Network of people who inject drugs and their contacts in the Transmission Reduction Intervention Project (TRIP) with component (pink) and nearest neighbor set (orange) for a participant (red). The full TRIP network displays individuals or nodes (green) and connections or edges (gray).

**Figure 6:**
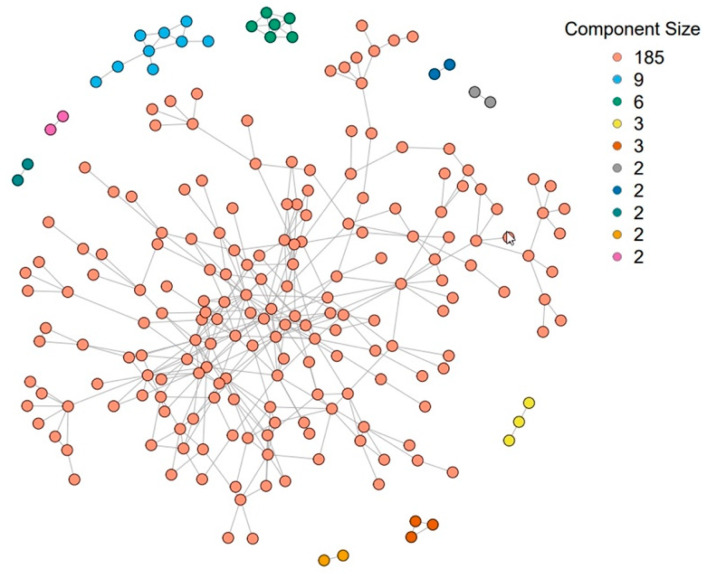
The Transmission Reduction Intervention Project (TRIP) network was used to analyze the spillover of a community alerts intervention on HIV risk behavior (*n* = 216). This network consisted of 10 connected components. The size of each component after using community detection to further divide the network into 20 components is {28, 26, 23, 19, 19, 18, 15, 12, 10, 9, 8, 7, 6, 3, 3, 2, 2, 2, 2, 2}.

Recent work considered nearest-neighbor IPW estimators to quantify the spillover effects of non-randomized interventions in a sociometric network study on subsequent health outcomes [56,57,65]. Two different IPW estimators were shown to have reasonable statistical performance in this setting, and a variance estimator was provided, allowing for overlapping spillover sets within network components. In this work, the spillover set is defined specifically to each participant and includes their nearest (first-degree) neighbors in the network (Figure 5). An exposure propensity score is used to account for cofounding with measured baseline covariates through inverse probability weighting and defined as the probability of exposure for the spillover set (an individual and their neighbors). That is, each individual is weighted by the inverse of the probability of their exposure and exposures for their spillover set. For these two estimators, no weights are applied to the edges. The first IPW estimator models the exposure propensity score using a mixed effects logit model with a random intercept to account for the correlation of exposures in the spillover set (Figure 5). This is an extension of the estimator in Liu et al. (2016) [65] with the spillover sets defined by the nearest neighbors for each individual in the network and assumes that the exposures for the individual and their neighbors are independent conditional on the exposure and covariates for the individual and their neighbors and the nearest neighbor random intercept. The second estimator uses two logistic regression models (one for the individual propensity score and one for the nearest neighbor’s propensity score) to estimate the exposure propensity score, applying the approach in Forastiere et al. (2021) [56] to an IPW estimator. This estimator assumes that the exposures in the spillover set are independent conditional on the exposures and covariates for the individual and their neighbors. This estimator may be preferable if the dependency among neighbors’ exposures cannot be attributed to a latent factor (i.e., random effect). Both estimators use the components as independent units in the network for the variance estimators used to obtain the standard error and corresponding Wald-type confidence intervals (Figure 6). A simulation study found reasonable finite-sample performance of the estimators with at least 50 components and an improvement in efficiency (i.e., smaller estimated variance) using the first IPW estimator, as compared to an approach to estimate the variance that assumed partial interference by network components [65].

Another area of the literature leverages targeted maximum likelihood estimation (TMLE) to assess the effects, including spillover of non-randomized interventions in networks [129]. TMLE is an estimation algorithm focusing on a target parameter of interest to allow for a more efficient and doubly robust estimator [130]. A sequence of recent work developed and improved a TMLE approach to estimate the causal effects of a non-randomized intervention in a network, including methods for dynamic (i.e., intervention assigns exposure as a deterministic function of covariates) and stochastic (i.e., intervention assigns exposure as a random function of covariates) interventions [129,131,132]. Ogburn et al. (2022) developed a TMLE approach to estimate causal effects in a single network that allows for dependence among the individuals in the network due to direct transmission and latent characteristics among the individuals [51]. Their work also includes a new central limit theorem for dependent data and interesting extensions that allow for interventions on the network structure. In their approach, a structural equation model is used to define the causal effects of interest. Their estimator is a targeted maximum loss-based estimator, which is shown to be (asymptotically) normal.

### 3.5. Methodological Considerations

Beyond these recent methods’ developments to assess spillover in networks, there are important considerations specific to causal influence in a network and approaches to handling missing data when evaluating spillover effects. The definition of causal influence allows the researcher to examine the assumptions under which influence aligns with network centrality measures. In evaluating spillover, missing information on outcomes, edges, and covariates threatens the validity of the analysis, and methodological approaches are currently available under the partial interference assumption.

#### 3.5.1. Causal Influence in a Network

Researchers from a wide range of fields are interested in evaluating the influence of individuals in a network. Particularly in public health, identifying the most influential individuals in a network is crucial for allocating resources or increasing intervention impacts. Network ties can be utilized to optimize health and behavioral improvements by intervening on only a subset of individuals—who can be considered “influential” individuals—in a network [16,133,134].

In many contexts, what researchers ultimately are looking for in influential nodes corresponds to a causal notion of influence, which is defined through the changes in collective outcomes given a fixed intervention level. For example, which nodes should we intervene with to have the largest causal impact across the entire network? However, much research on identifying the most influential individuals in a network has focused on an observed feature of networks to understand influence, either extracting specific features of networks (e.g., using an adjacency matrix) or modeling specific diffusion processes (e.g., assuming a homogeneous transmission probability from one node to its adjacent nodes). Only a small number of studies in the related literature have made these assumptions about diffusion processes explicit. As a result, there could be a critical discrepancy between what is actually measured and what should be measured in causal influence research in networks. Important work remains to determine under which assumptions commonly used centrality measures, such as degree and betweenness centralities, align with a causal definition of influence. One possible approach is to define the influence of individuals on a network as a causal effect on collective outcomes using a potential outcomes framework.

Despite its direct relation to many research questions, the identification of causal measures of influence is challenging compared to many other centrality measures. Especially when we observe a single snapshot (instead of multiple replicates) of a network, we need to rely on parametric assumptions about the intervention and its relationship to the collective outcomes observed in a network [71,127]. Unfortunately, many of these parametric model-based methods will result in biased influence estimates when unmeasured confounders are present, and the models are misspecified. Therefore, future work is needed to develop a randomization study designed to learn about the influence of individuals in a network. Researchers may also consider using more flexible centrality measures, such as random-walk betweenness centrality rather than the standard betweenness centrality [135].

#### 3.5.2. Missing Data in Networks and Evaluation of Spillover

In network-based studies aimed at the evaluation of spillover, missing information presents a challenge for statistical inference, including missing outcomes, covariates (used to control for confounding), missing edges, and missing information due to network sampling. The type of missingness is usually classified into three categories [136]: “Missing Completely at Random” (MCAR), the probability of missingness does not depend on either the observed or unobserved data; “Missing at Random” (MAR), the probability of missingness only depends on the observed data; and “Missing Not at Random” (MNAR), the probability of missingness also depends on the unobserved data. Due to the large amounts of missing data, it is of great practical interest to incorporate the missing data in the analysis of causal inference in studies evaluating spillover.

If we are willing to assume MAR, several methods depend on the observed data, including maximum likelihood, multiple imputation, fully Bayesian, and inverse probability weighting [137,138]. In network studies, some approaches assume MAR for missing edges or participant characteristics, including an exponential random graph modeling (ERGM) approach focused on missing dyads (i.e., pairs of participants) [139], simple imputation approaches using unconditional mean/distribution, preferential attachment, hot deck [140], a Bayesian approach for missing information on edges [141], a likelihood approach to address missing information due to network sampling [142], and a necessary and sufficient condition of consistency for models that belong to exponential families of stochastic processes, including ERGMs [143]. In the presence of spillover with outcomes missing at random, recent work has developed methods for right-censored (i.e., survival) outcomes that use an inverse probability of censoring weight to account for missing survival outcomes estimated with a survival model, such as a proportional hazards frailty models or accelerated failure time model [94,95]. These approaches in the presence of spillover typically assume MAR and spillover sets defined by groups of participants (i.e., partial or generalized spillover sets). These estimators could be used in studies that either did not collect information on the connections in the network (e.g., cohort study or randomized trial) or, if a network study, edge information is not used in the estimation. In the presence of missing edges, many existing methods assume MAR, and are thus not applicable if edges are MNAR. For example, PWUD might not be willing to disclose illicit substance use partnerships due to stigma concerns, and consequently, the edges are likely to be MNAR. Future work is needed to address MNAR edges and participant characteristics in network-based studies, possibly with a joint modeling framework.

## 4. Illustrative Example: The Transmission Reduction Intervention Project in Athens, Greece

### 4.1. Description of TRIP

The Transmission Reduction Intervention Project (TRIP) in Athens, Greece, included people who inject drugs (PWID) and their HIV risk networks focusing on people who had recently been infected by HIV, as indicated by an assay developed for that purpose or according to the testing history of the participants [37,144]. The concept was that TRIP participants who acquired HIV recently would be more likely to be surrounded by others who were also possibly recently infected, who either infected the participants or were infected by them, or shared a recent “ancestral infector”. Consequently, tracing the risk networks of recently infected individuals could be an efficient way to identify other undiagnosed but infectious persons who need immediate care. TRIP started with PWID who had participated in the RDS ARISTOTLE study in Athens and were initially referred and recruited into TRIP if they were found to be recently infected with HIV [145]. Each recently diagnosed individual (i.e., recent seed) was asked to identify their sexual and drug-use partners or those present at drug-using venues when the participants engaged in risky behaviors at those locations. These contacts were then recruited and asked to identify, in turn, their sexual and drug-use partners, who were also recruited, and their connections to other individuals in the study were ascertained. If any of these partners were identified as recently infected with HIV, then their contacts and the contacts of their contacts (i.e., two waves of contact tracing) would be recruited as well, and their contacts were ascertained [146]. In addition, longer-term HIV-infected individuals (long-term control seeds) were also recruited, and their network contacts were traced based on the same procedure described above. Finally, HIV-negatives at baseline served as a control group without any further work by the TRIP staff to identify their HIV risk partners. All TRIP participants were interviewed using a questionnaire containing items on demographics, sexual and injection behaviors/partners in the prior six months, drug treatment, and antiretroviral treatment. A second round of interviews and HIV testing followed six months after the baseline.

### 4.2. Description of TRIP Network

The abovementioned information was used to create a final observed network where each recruited individual was linked to all other individuals who named them as a contact or were named as a contact by them, regardless of recruitment order. TRIP recruited 356 participants (nodes) with 542 direct ties (edges) between them. The average network degree was 3.045, and the overall network density was 0.0086 [17]. The network in TRIP consisted of a large component (*n* = 241), where all nodes are directly or indirectly connected, a few small components with 2 to 10 individuals each, and some isolates (*n* = 79) [17]. The average distance between reachable nodes in the large component was 4.9. The large component included 172 two-core members (48.3%), 100 three-core members (28.1%), 65 four-core members (18.3%), and six five-core members (1.7%). There were 246 three-cliques in the two-core of the large network component, and 210 three-cliques with 95 (26.7%) members in the three-core [17]. A three-clique is a subset of nodes where each node is connected to the others in the subset at a distance of three.

### 4.3. TRIP Findings

In accordance with the hypothesis formulated by the TRIP research group, the proportion of recently HIV-infected people in the network of recent seeds was indeed much larger (i.e., three times) than that in the network of long-term infected controls [37]. Moreover, the TRIP staff, using a combination of approaches to improve case management (information and instrumental support, multiple reminders, multiple and consistent efforts to overcome administrative obstacles), was achieved to connect almost 87% of the recently HIV-infected PWID participants into care; 77% of those began antiretroviral treatment, and eventually 89% had very low viral load levels [147]. The data analyses also showed that the behavioral patterns reported by the participants at the follow-up interview of TRIP were much safer than those reported at the start of the project [148], while the project was not associated with any adverse events in terms of the mental health of the participants or any TRIP-related stigmatizing experiences [146,149]. Instead, there was some evidence that HIV-related support increased significantly over the course of the project [149]. Finally, valuable associations between network structure and HIV prevalence and behaviors/norms were identified. For instance, HIV prevalence was significantly higher in the large component, the two-core and three-core of the large component, and the three-cliques of the cores [17]. In terms of network norms, the analyses showed that a safe behavioral pattern (use of unclean cooker/filter/rinsing water was never encouraged) was significantly less normative among individuals who were contacts of TRIP participants in the two-core than among network contacts of TRIP participants outside the two-core [17]. Of interest, the analyses of TRIP data also showed significant associations between drug injection-related norms and risky behaviors [31]. For example, TRIP participants who recalled that they were encouraged by their drug partners to use a contaminated syringe were more likely to report that they shared syringes than those who did not recall such an encouragement.

### 4.4. Spillover Findings in TRIP

Beyond the impactful findings described above, both in terms of effectiveness and safety, interventions in TRIP could also have spillover effects. This became apparent when appropriate analyses were performed (i.e., using IPW estimators where the spillover set consisted of the nearest neighbors of the participants, i.e., those with a first-degree connection with a particular participant) [57,65]. The TRIP staff distributed community alerts, which were paper flyers provided to participants and posted in locations frequented by members of the local PWID community [6]. The community alerts told people that someone in their “social proximity” had recently acquired HIV, which means that others are perhaps infected and may not know it. They urged people to take a test for undiagnosed early HIV infection and to be careful to avoid HIV-related risks for the next six months. After excluding isolates and those with missing data, a total of 216 participants were included in this analysis. In total, 25 (12%) of the 216 participants were exposed to community alerts [57].

Given the smaller number of components in the study, the second IPW estimator may be preferable due to confidence interval coverage closer to the nominal level in a statistical simulation study [57]. For the second IPW estimator with 10 components (see Section 3.4), the estimated unstabilized weights ranged from 0.003 to 28.24 for the intervention coverage levels considered in the analysis (i.e., 20%, 30%, 40%, 50%). Assuming that 50% of the nearest neighbors of a participant receive the community alerts, the direct effect (on the risk difference scale) of community alerts on sharing injection equipment was −0.21 (95% confidence interval (CI) = −0.56, 0.15). This means that we expect 21 fewer reports of risk behavior per 100 participants if an individual receives an alert compared to if an individual does not receive an alert with 50% intervention coverage (i.e., 50% of neighbors receiving alerts). In addition, the analyses showed spillover effects (risk difference = −0.02; 95% CI = −0.04, −0.01) for community alerts coverage of 50% versus 30% for nearest neighbors. In other words, we expect two fewer reports of risk behavior per 100 participants if a participant does not receive an alert with 50% intervention coverage compared to only 30% intervention coverage. Most estimates of the risk differences were protective. In this study, the proportion of an individual’s neighbors exposed to community alerts from the study team, in addition to an individual’s exposure to community alerts, reduced HIV risk behavior. This analysis highlights the importance of understanding the suite of effects in the presence of spillover to evaluate intervention mechanisms and inform the development of future interventions among PWUD.

## 5. Software for Spillover in Networks

In an observational study with partial interference (e.g., assuming the spillover set is comprised of the network components), an existing R package inferference [150] can be used for unstabilized IPW estimators, and the stabilized interference package (v0.0.2.9200) [151] is available for stabilized IPW estimators. For the ego-network network randomized design, example code to assess spillover using mixed effects models in SAS is provided in the supplement (Web Appendix 4) of Buchanan et al. (2018) [44]. For evaluation of spillover in network studies, there are programs and software available for some settings. For the two IPW estimators for spillover of a nonrandomized intervention in a sociometric network, an R function with a toy data set is available on GitHub https://github.com/uri-ncipher/Nearest-Neighbor-estimators. For a TMLE approach to assess causal effects with a time-invariant intervention in network data, the tmlenet R package is available https://rdrr.io/cran/tmlenet/man/tmlenet-package.html. For the evaluation of causal influence in networks, example code is provided on GitHub https://github.com/uri-ncipher/CausalInfluence.

## 6. Discussion and Future Directions

This paper provides a tutorial about approaches to assess spillover in network-based studies among people who use drugs. Consideration beyond individual risk behavior for HIV prevention is required because these individuals are part of sexual, social, and/or drug-use networks [4,5,6,7,8]. Network-based interventions offer much promise of reducing HIV transmission and bolstering harm reduction measures among PWUD and their networks [6,32,37]. While interventions delivered and evaluated among individuals can reduce HIV transmission risk, the delivery of interventions in networks has the potential for substantial benefit beyond the exposed individual. This is broadly known as spillover (i.e., interference or dissemination) and occurs when one participant’s exposure affects another participant’s health outcome. Participants in intervention studies are often part of underlying networks even if networks are not explicitly measured or a focus of the study. Importantly, spillover likely occurs in these networks and is largely ignored in the evaluation of HIV interventions, often underestimating the full intervention impact.

Study designs in the network setting include randomized designs, ego-network randomized designs, respondent-driven samples, and sociometric network designs. Methods for each of these designs are in various stages of development. This tutorial notes the limited use of network-randomized designs to evaluate interventions among PWUD, as well as the limitations of methods to assess spillover in RDS designs due to limited multivariable methods needed to adjust for covariates to address confounding in causal inference, as well as challenges due to the RDS sample not representing the full network. New methods for ego-network randomized designs and sociometric designs show promise for impactful real-world applications and continued methods development for spillover evaluation in these network study designs. An evaluation of spillover can be framed by considering the study design and then defining the spillover set and often the corresponding exposure mapping. In recent work, a participant’s nearest (first-degree) neighbors were used to define the spillover set [57], but the spillover set could be defined to include second- or third-degree neighbors or even the entire component or full network.

Numerous future study design and methodological problems exist for network-based studies among PWUD. In terms of study design, the sampling of networks is often complex, resulting in selection bias and open questions about defining the population in which inference is conducted. Future work could improve methods for the generalizability of spillover effects [88,91], possibly leveraging approaches to generalize mediation effects [152], as well as improving weighted generalized estimating equations with spectral clustering in order to address sampling bias in a network study with an insufficient number of (unconnected) network components [153]. Although there is evidence that PWUD accurately report information, such as name and age, for their network members [154], improved methods are needed for approaches to ascertain the edges or connections for individuals in network-based studies [59,155,156], such as entity resolution and alter-ego matching algorithms. In ego-network randomized designs, improved approaches to collect information on contamination from the intervention to control networks and causal inference methods to detect and adjust for contamination are needed [108]. Furthermore, the outcome of interest might be missing due to participant loss to follow-up in network-based studies with longitudinal follow-up. For example, 18% of TRIP participants were lost to follow-up by the six-month visit. Future work is needed for methods to address study dropout and other missing data issues in networks, particularly for the information that is not missing at random, including missing connections or edges. Evaluating the impact of unmeasured confounding is important because this assumption cannot be tested, but only assessed through sensitivity analyses. Sensitivity analyses in the presence of spillover are available for two-stage randomized trials with clustering features [157]. Additional work is needed to assess the bias of unmeasured confounding when evaluating spillover in network-based studies. Network dependence or network autocorrelation refers to statistical dependence within a variable due to network edges [62,158,159]. The anti-conservative inference (i.e., the variance underestimation problem) due to network-dependent samples is relatively well known. However, careful attention is still needed when researchers draw an associational or causal inference between two (or more) variables based on network-dependent samples. The problem of spurious associations has been discussed in temporal dependence [160,161], but there has been very little literature in network contexts even though associations are frequently measured in network-based studies among PWUD, and this is an important area for future work. In addition, if the spillover set actually included two-degree neighbors or other sets of individuals in the network, the nearest-neighbor interference assumption would not be valid. We recommend extensions to the methods that consider alternative definitions of the spillover set in the network and assess the extent to which results change under alternative assumptions on the spillover sets [92,93]. When information on the true social relationships is only available for a subset of the sample, validation study/main study bias correction methods could be used to correct for the bias resulting from the definition of spillover sets based on the available information on randomization clusters or geographical distances. Another interesting future direction is the incorporation of edge weights into this method to reflect variations in the strength of connections relevant for spillover [162,163].

## 7. Conclusions

Interventions delivered in networks and network-based interventions have benefits beyond those observed for individuals who receive the intervention themselves. People who use drugs and are part of networks are thus likely to benefit from the exposure of their network contacts through spillover mechanisms, even if they are not exposed to an intervention themselves. Methodology to quantify the effects of network-based interventions with spillover has been increasingly developed, though it has largely yet to reach a wider public health audience beyond statistical methodologists. However, understanding the full impact of interventions is a prerequisite for fair and comprehensive project evaluation, policymaking, and designing and funding new research. This tutorial summarizes relevant methodology and clarifies terminology, definitions, and assumptions to narrow the gap between new methods development and the large groups of non-methodologists in the infectious disease domain, including policymakers, and medical and public health experts.

## Figures and Tables

**Table 1:** Related terminology for spillover effects in a network-based study.

Recommended Term	Alternative Terms	Definition	Meaning
Direct	Individual	Contrasts average (potential) outcomes under exposure versus no exposure with a fixed treatment allocation strategy in the spillover set	Intervention effect on the exposed individuals while holding the treatment allocation constant in the spillover set
Spillover	Indirect, Disseminated	Contrasts average (potential) outcomes if a participant was not exposed comparing two treatment allocation strategies in the spillover set	Intervention effect on the unexposed individuals comparing two different treatment allocation strategies in the spillover set
Composite	Total	Contrasts average (potential) outcomes if a participant was exposed under one treatment allocation strategy in the spillover set to not exposed under another allocation strategy	Maximal intervention impact; effect of both the individual exposure and treatment allocation strategy in the spillover set
Overall	Crude	Contrasts average (potential) outcomes under different treatment allocations for the index individual and their spillover set	Marginalizes over individual intervention; Effect of the group-level allocation strategy

## Data Availability

The TRIP datasets are available upon reasonable request to the corresponding author subject to approval by the TRIP investigators. The HPTN 037 study data sets are publicly available and can be requested from the Statistical Center for HIV/AIDS Research and Prevention through the ATLAS Science Portal at https://atlas.scharp.org/cpas/project/HPTN/.
